# Study on Mechanical Properties and Degradation Behavior of Magnesium Alloy Vascular Clip

**DOI:** 10.3390/jfb14100501

**Published:** 2023-10-09

**Authors:** Hongxu Zhang, Ming Gao, Xiaoying Tian, Dali Cao, Lili Tan

**Affiliations:** 1 Materials Science and Engineering, Shenyang University of Chemical Technology, 11# Street Shenyang Economic & Technology Development Area, Shenyang 110142, China; zhang202394@163.com; 2 Institute of Metal Research, Chinese Academy of Sciences, Shenyang 110016, China; mgao16b@imr.ac.cn (M.G.); xytian20s@imr.ac.cn (X.T.)

**Keywords:** finite element modelling, biodegradable Mg alloy, vascular clip, structural design, corrosion resistance

## Abstract

The Mg alloy vascular clip has biodegradability and good biocompatibility, which can improve the convenience and safety of clinical application. However, the Mg alloy vascular clip also has some disadvantages, such as an unreasonable structure design and a degradation rate which is too fast. In this study, the process of clamping blood vessels with a biodegradable Mg alloy (Mg-Zn-Nd-Zr and Mg-Zn-Nd) general V-type vascular clip was simulated by finite element simulation software (Abaqus). A new type of vascular clip, the P-type vascular clip, was analyzed and investigated through simulation. The differences between Mg alloy vascular clips of V-type and P-type were analyzed by finite element simulation. In addition, the effects of Zr element on the mechanical properties and corrosion resistance of P-type vascular clips were also investigated to improve the mechanical stability. The results show that during the V-type vascular clip closure of Mg-Zn-Nd-Zr alloy, this clip has some problems, such as uneven distribution of blood vessel stress, crevices in blood vessels and stress concentration. The improved P-type vascular clip has uniform closure, and there is no gap in the blood vessel, which can effectively avoid stress concentration. The improved P-type vascular clip is well closed and can effectively avoid stress concentration. The corrosion resistance of the Mg-Zn-Nd-Zr alloy P-type clip was better than that of the Mg-Zn-Nd alloy P-type clip (degradation rate of 2.02 mm/y and 2.61 mm/y on the 7th day, respectively). Mg-Zn-Nd-Zr alloy The P-type vascular clip remained closed even on the 7th day, which could meet the requirements of clinical application.

## 1. Introduction

The vascular clip is mainly used in minimally invasive surgery represented by laparoscopic surgery, and its adequate hemostasis is particularly important for surgery success and reduction of post-operative complications [1,2]. At present, the vascular clip most widely used in the clinic is the titanium (Ti) clip. The undegradable Ti alloy clip will remain in the human body for a long time. It will interfere with computed tomography (CT) or nuclear magnetic resonance imaging (MRI) scanning, and even cause allergic reactions. These shortcomings will cause a certain confusion and influence the follow-up treatment and psychology of patients [3,4]. Biodegradable magnesium (Mg) and its alloys have good biocompatibility, mechanical stability and ductility. Therefore, the alloy vascular clip has great application potential [5]. However, some weaknesses, such as unreasonable structural design or local corrosion caused by galvanic corrosion, limit its application [1,6,7]. 

In terms of structural design, the vascular injury degree caused by the vascular clip is closely related to the pressure and diameter of the clip, the vascular diameter and the health status of the blood vessel. The clamping force of the vascular clip also has a certain influence on the phenomenon [8]. In addition, the structural design also has a certain impact on the degradation behavior of Mg alloy. Wu et al. [9] found that Mg alloy surgical staples would be corroded rapidly at the position of stress concentration after being anastomosed, resulting in premature failure. Therefore, the structural design of the Mg alloy vascular clip is key. However, there is little research on the structural design of biodegradable Mg alloy vascular clips at present. Most of the designed structures are relatively simple, mainly V-type vascular clips. Although a series of in vivo and in vitro studies have preliminarily verified the feasibility of the Mg alloy vascular clip, it still has some disadvantages to overcome. The leakage and rebound of the V-type vascular clip will cause the clip to not be fully closed, resulting in space between the blood vessel [1,10]. Also, residual stress in the arc position at the bottom of the vascular clip will induce premature failure [11]. These disadvantages will ultimately affect vessel closure and the mechanical stability of the vascular clip.

As for corrosion resistance, the clamping of the vascular clip requires a stable environment and closing time. The diameter of the vascular clip is too small (only 1 mm in thickness). Once local corrosion occurs, the Mg alloy vascular clip will fail in advance. Therefore, the corrosion resistance and degradation behavior of Mg alloy are another key performance factor for its application as a vascular clip. Alloying, microstructure control and surface modification are effective ways to improve the corrosion resistance of Mg alloys [12,13]. The corrosion resistance of magnesium alloy with 0.41% Dy element is the best, but the high content of rare earth elements would affect the biocompatibility of Mg alloy [14]. Li et al. [15] proposed the use of poly-butylene adipate-co-terephthalate (PBAT) coating with high elongation to improve the corrosion resistance and biocompatibility of Mg alloy vascular clips. Although surface modification can effectively improve the corrosion resistance of magnesium alloys, there is still a problem as to whether the coating can maintain a certain integrity after vascular occlusion. 

In this study, Mg alloy vascular clips with different structures and compositions were designed and investigated. The stress concentration was alleviated by optimizing the vascular clip arc end. The design of the top clip was used to ensure the closure of the vascular clip and solve the problem of rebound. To balance the relationship between biocompatibility and corrosion resistance, alloying with a low elements content was carried out, and their feasibility as vascular clip materials was studied. The stress distribution of Mg alloy vascular clips with different structures before and after closure was investigated by Abaqus 2022 (SIMULIA, Bota, RI, USA). In addition, the mechanical properties and in vitro corrosion resistance of Mg alloy vascular clips with different materials were tested and analyzed.

## 2. Material and Methods

### 2.1. Vascular Clip Design 

**Figure 1:**
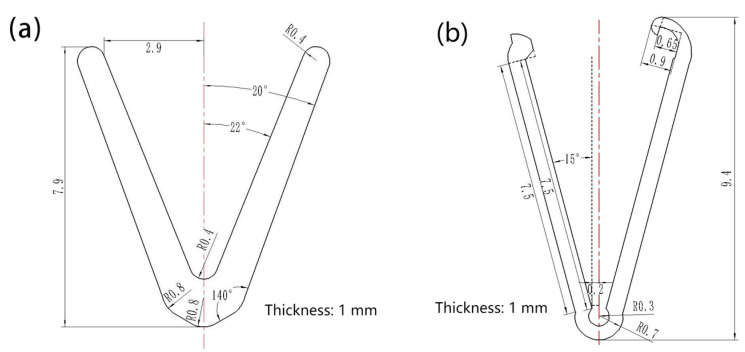
Structural size of vascular clip ((**a**): General V-type clip [16], (**b**): Improved P-type clip).

Figure 1 shows the structure and size of the vascular clip. Figure 1a is the general V-type vascular clip [16] with an overall size of 7.9 mm and a thickness of 1 mm. Figure 1b is the improved vascular clip. The improved P-type vascular clip mainly consists of three parts, the bottom arc section, middle clip arm and top clip. The P-type overall size of the vascular clip is about 9.4 mm, and the thickness is 1 mm.

### 2.2. Finite Element Simulation Analysis of Magnesium Alloy Vascular Clip

Solidworks 2022 (Dassault Systemes, Concord, MA, USA) was used to establish the geometric model of the vascular clip and blood vessel. Abaqus 2022 was used to simulate and analyze the stress-strain and structural deformation of the vascular clips. Based on the simulation analysis of the general V-type vascular clip, the P-type vascular clip is designed to make it more convenient, safe and reliable. The advantages and disadvantages of different materials and structures and their influence on application were discussed by comparing the finite element analysis results. 

Arterial segments were used in the Abaqus vascular model; the length is 10 mm, the blood vessel diameter is 2.5 mm, and the artery wall is 0.25 mm. The artery wall is composed of inner, middle and outer tissues, of which mainly the middle and outer tissues are subjected to pressure. In addition, the mechanical properties of arteries depend on the mechanical properties of collagen fibers, elastic fibers, and smooth muscle components in the tissue, which are the factors that produce stress relaxation and elastic hysteresis of the artery wall, and these factors add up to form the super-elasticity of the artery wall. The main mechanical properties of the artery wall include anisotropy, heterogeneity, nonlinearity and incompressibility [17]. Table 1 shows the main parameters of the Holzapfel arterial wall HGO constitutive model for the Abaqus vascular model [18]:

In Table 1, C_10_, K_1_ and K_2_ are dimensional parameters of blood vessels, which can affect the torsional strain energy and volumetric strain energy. These dimensional parameters play an important role in the stress distribution of blood vessels. Besides, φ is the material parameter that can influence the fiber group direction and fiber group distribution density in the material. Therefore, C_10_, K_1_, K_2_, φ are four essential parameters for different organizations [17,18]. In this design, the material parameters of the above four kinds of blood vessels are fixed during the simulation process.

### 2.3. Magnesium Alloy Preparation

The cast Mg-Zn-Nd-Zr and Mg-Zn-Nd alloy ingot with a diameter of 85 mm were processed into 100 mm long blanks. Since zinc has the largest solubility in Mg at 325 °C, and the diffusion rate of rare earth elements in magnesium alloys is slow. 325 °C × 10 h were selected as the homogenization process parameters. After homogenization, the ingot was turned into a bar with a diameter of 80 mm and the surface oxide was removed. The cast bar material was preheated at 390 °C for 2 h, and the extrusion Mg-Zn-Nd-Zr and Mg-Zn-Nd alloys were extruded into bars (10 mm in diameter) with an extrusion speed of 10 mm/s [19]. Table 2 shows the composition and content ratio of Mg-Zn-Nd-Zr and Mg-Zn-Nd rods of Mg-based materials with an extruded diameter of 10 mm. 

### 2.4. Preparation of Mg Alloy Vascular Clip 

The Mg alloy vascular clip was processed according to the dimensions in Figure 1 through mechanical processing. Then it was placed in the acid-washing solution (HNO_3_: Absolute ethanol = 1:99, 0.3 g Methenamine) for 10 min for ultrasonic washing, so as to remove the oxide layer caused by mechanical processing. After pickling, the sample was ground to 1 mm thickness with # 2000 SiC paper (Damao chemical reagent factory, Shenyang, China). Finally, the sample was polished, ultrasonically washed in alcohol for 5 min, and the clip was dried in an incubator (Damao chemical reagent factory, Shenyang, China) for in vitro immersion and other experiments.

### 2.5. Microstructure Characterization 

The treated vascular clip was polished with SiC paper for # 2000, # 3000 and # 5000. After the surface was smooth and bright, the sample surface was polished with 2.5 μm and 0.5 μm diamond polishing paste in turn. During the polishing process, absolute ethyl alcohol was continuously added until the surface was bright and with no scratches. Then the samples were treated with etching agent (6 g picric acid, 10 mL glacial acetic acid, 10 mL distilled water and 70 mL alcohol). After the sample was etched for 13 s, the sample surface was cleaned with deionized water and anhydrous ethanol, and metallographic observation was carried out after air drying. The microstructures of Mg-Zn-Nd-Zr and Mg-Zn-Nd alloys were observed by metallographic microscope (Zeiss ZM-1), and the metallographic optical microscope (Zeiss ZM-1) (Carl Zeiss AG, Oberkochen, Germany) with a magnification of 500:1. 

The microstructures of Mg-Zn-Nd and Mg-Zn-Nd-Zr materials were analyzed by SSX-550 scanning electron microscope (SEM, Shimadzu Corporation, Kyushu, Japan) with an operating voltage of 20 kV. The phase composition of Mg-Zn-Nd-Zr and Mg-Zn-Nd alloy vascular clips before and after immersion was analyzed by D/max 2500X-ray diffractometry (XRD, Marvin Panaco, Almelo, The Netherlands). The radiation source was Cu target K_ɑ_ rays. The working voltage was 40 kV, the current was 40 mA, and the scanning rate was 10°/min. Jade 6.0 software (Sun, CA, USA) was used to analyze the phase composition of XRD data. 

### 2.6. Mechanical Properties Test 

According to GB/T 16865-2013 [20], Mg alloy samples were used for tensile testing with the gauge length and width of 25 mm × 6 mm was processed by molybdenum wire cutting. After processing, the sample surface oxide layer was polished with # 2000 SiC paper. At room temperature, the ZwickZ050 universal machine (Zwick, Ulm, Germany) was used to carry out unidirectional tensile test with a speed of 1 mm/min. Three parallel samples were used for each sample to ensure accurate and reliable results.

### 2.7. In Vitro Degradation Analysis 

Because the composition of SBF is closest to that of blood, SBF solution was selected to simulate human body fluids, for which the components are shown in Table 3. The components of SBF solution are added in sequence according to the serial number in the table. The concentration of HCl solution is 1 M/L. The pH of the solution is adjusted to 7.45 with 0–5 mL of 1 M/L HCl solution. The vascular clips were divided into open clips and closed clips, setting 3 parallel samples for each group and adding 3 blank samples. After soaking in SBF for 7 days, the pH variation of these 7 days was recorded. The weight loss at 1 day, 3 days, 5 days and 7 days was recorded to obtain the degradation rate. After soaking, the corrosion products of the vascular clip were cleaned for 1 min at 20–25 °C by acid-washing solution (200 g CrO_3_, 10 g AgNO_3_, 20 g Ba[(NO_3_)_2_]) to weight. The corrosion morphologies of the Mg alloy vascular clip before and after corrosion were observed by Scanning electron microscope (SEM SSX-550) at the working voltage of 20 kV. The calculation formula for the corrosion rate is (1):

(1) $$V=\frac{K\times W}{A\times T\times D}$$

where V is corrosion rate (mm/y), K is corrosion rate constant, 87,600, W is mass loss (g), A is total surface area of sample (cm^2^), T is soak time (h), and D is material density (g/cm^3^).

## 3. Results

### 3.1. Microstructure Characterization 

**Figure 2:**
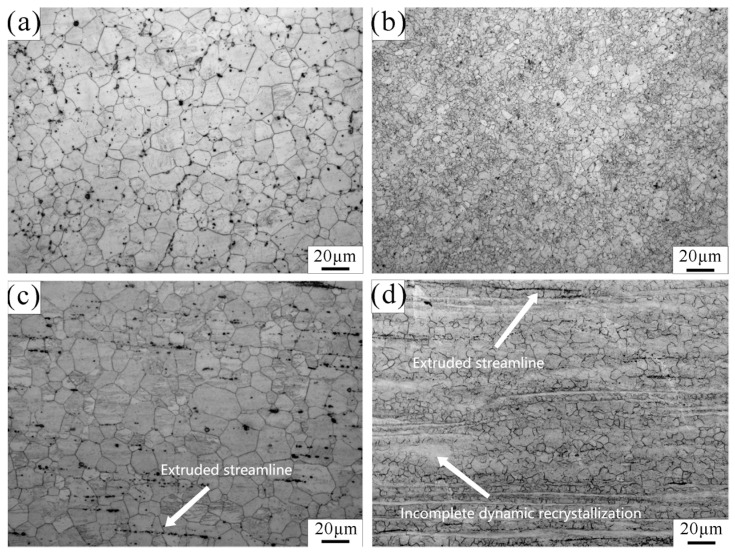
Optical microstructure of. Mg-Zn-Nd alloy (**a**,**c**) and Mg-Zn-Nd-Zr alloy (**b**,**d**). ((**a**,**b**): Transverse metallographic diagram; (**c**,**d**): Longitudinal metallographic diagram).

Figure 2 shows the optical microstructure and second phase distribution of Mg-Zn-Nd and Mg-Zn-Nd-Zr alloys. Figure 2a,b are the transverse planes of the extrusion process. Figure 2c,d are the Longitudinal planes of the extrusion process. It can be seen that the extruded Mg-Zn-Nd alloy exhibits obvious characterization of dynamic recrystallisation. The grain distribution was relatively uniform, with an average grain size of 21.71 μm, while extruded Mg-Zn-Nd-Zr alloy undergoes incomplete dynamic recrystallization. The alloy structure consists of un-recrystallized grains and recrystallized grains, with an average grain size of 14.31 μm. Both materials had obvious extrusion flow lines. The elongated grain and extruded streamline are marked in Figure 2.

**Figure 3:**
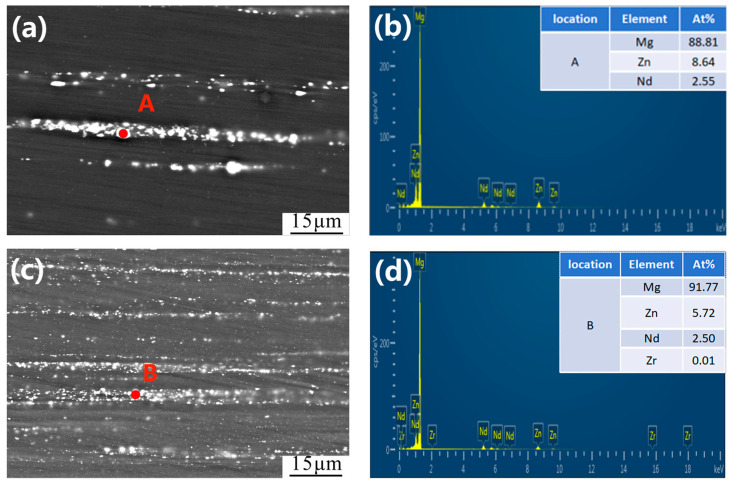
SEM micrograph and EDS spectrogram of. Mg-Zn-Nd alloy (**a**,**b**) and Mg-Zn-Nd-Zr alloy (**c**,**d**) of longitudinal planes and EDS results of position A (**c**) and B (**d**).

Fine second phases with dispersed distribution can be observed in Figure 3a,c. The dimensions of Mg-Zn-Nd and Mg-Zn-Nd-Zr alloys are about 2 μm and 1 μm. Three kinds of second phase with different shapes and sizes were distributed along the extrusion direction. The EDS results in this study only focus on second phases with larger dimension, because part of the second phase is too small to be detected. According to the EDS results of the two alloys in Figure 3b,d, it was confirmed that the second phase of Mg-Zn-Nd alloy contains Mg, Zn and Nd elements, while Mg-Zn-Nd-Zr alloy contains Mg, Zn, Nd and Zr elements. The different EDS results of second phases in the two alloys may be induced by the segregation of Zr around the second phase. Based on previous studies, it can be determined that these fine globular second phases are magnesium zinc phases, the small elliptic second phases are the Mg_12_Nd phase, and the largest one is the T (MgZnNd) phase [21]. To illustrate the amount of second phase in the alloy, the volume fractions were calculated by Fiji software (2.3.0, National Institutes of Health, Bethesda, WA, USA). The volume fractions of all second phases in Mg-Zn-Nd-Zr and Mg-Zn-Nd alloys were 9.33 ± 0.5% and 5.73 ± 0.4%, respectively.

**Figure 4:**
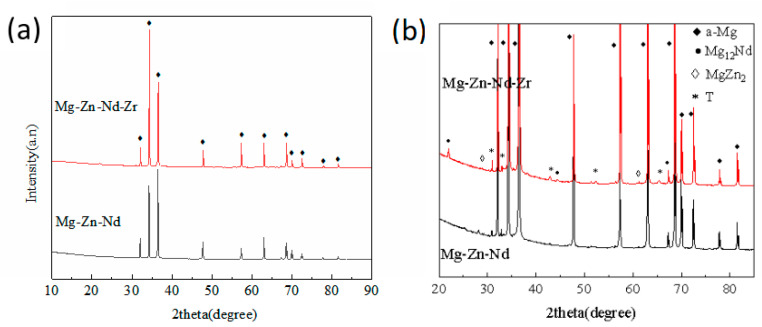
XRD patterns (**a**) and magnified images (**b**) of Mg-Zn-Nd alloy and Mg-Zn-Nd-Zr alloy.

The XRD analysis was conducted to further determine the composition of the second phase (Figure 4). Extruded Mg alloy has strong texture orientation, and the number of second phases in the alloy is very limited. The intensity of α-Mg peak intensity in Figure 4 is much higher than that of the second phase. Therefore, only α-Mg peak can be observed in Figure 4a with lower magnification. To make sure the peak of second phases is more obvious and confirm the type of the second phases, XRD results were magnified to higher magnification (Figure 4b). Based on XRD results and microstructure analysis, it can be determined that the type of the second phase in the two alloys is the same, Mg_12_Nd, MgZn_2_, and T (MgZnNd) [22].

### 3.2. Mechanical Properties 

**Figure 5:**
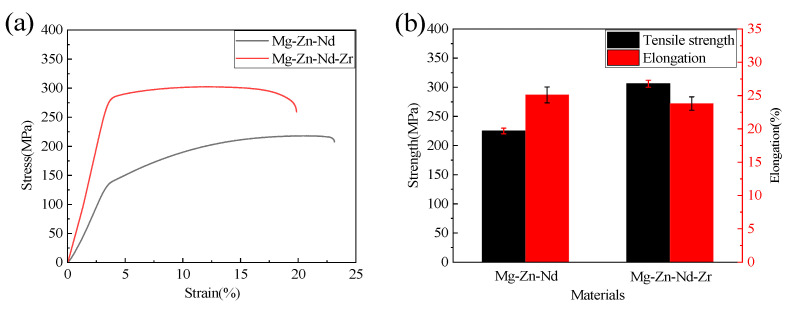
Mechanical properties of Mg-Zn-Nd alloy and Mg-Zn-Nd-Zr alloy. ((**a**): Stress–strain curves; (**b**): Tensile strength and elongation).

Figure 5 shows the stress–strain curves and mechanical properties of extruded Mg-Zn-Nd-Zr and Mg-Zn-Nd alloys. The ultimate tensile strength of Mg-Zn-Nd-Zr (305.95 ± 5 MPa) was 35.99% stronger than that of Mg-Zn-Nd (224.98 ± 5 MPa), while the plasticity of Mg-Zn-Nd-Zr (25.1 ± 1.2%) was 7.61% lower than that of Mg-Zn-Nd (23.8 ± 1%). 

### 3.3. Finite Element Analysis of Mg Alloy Clip 

**Figure 6:**
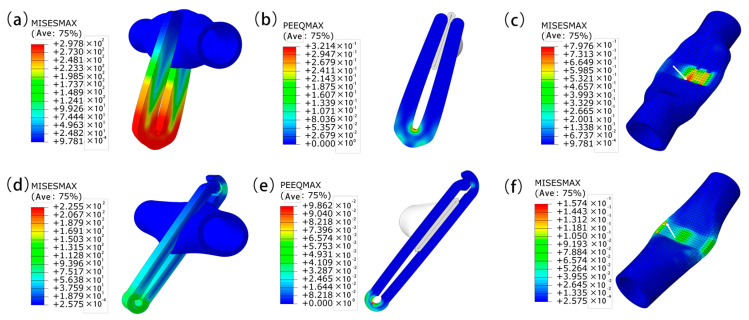
Stress–Strain analysis of general V-clips (**a**,**b**), P-clips (**d**,**e**), V-clip vessels (**c**), P-clip vessels (**f**) using Abaqus analysis.

Figure 6 shows the distribution of stress and maximum equivalent plastic strain (PEEQMAX) for the Abaqus simulation analysis of general V-type and improved P-type vascular clips. To ensure the accuracy of the results, the closest distance between the two arms of the two vascular clips during the closure phase should be consistent. Figure 6a shows the stress cloud diagram of the general V-type vascular clip. The stress concentration of the V-type vascular clip was located in the arc section at the bottom of the vascular clip, with a maximum stress of 297.8 MPa. Figure 6b is a cross-section cloud diagram of the V-type maximum equivalent plasticity (PEEOMAX). The PEEOMAX of the vascular clip had a bottom arc of 0.3214. Figure 6c shows the stress cloud diagram of the V-type blood vessel. The force on the blood vessel and the deformation at different positions were uneven. The maximum stress point of the blood vessel was the clamping position at the top of the vascular clip arm, with a maximum stress of 0.7976 MPa. The closure of blood vessels were uneven, which is caused by the structure of the vascular clip. The application of V-type vascular clips might lead to problems such as uneven closure of blood vessels and spaces in blood vessels. Therefore, the structure of the vascular clip has been improved. 

Figure 6d shows the stress cloud diagram of the improved P-type vascular clip. The stress concentration of the P-clip was located in the arc section at the bottom of the vascular clip, with a maximum stress of 221.8 MPa, which was 25.52% less than the maximum stress of the V-type vascular clip. Figure 6e is a cross-sectional cloud diagram of the P-type clamp PEEOMAX. The PEEOMAX of the vascular clip was 0.0928 at the bottom arc, and there were no spaces in the closure of the blood vessels. Figure 6f shows the stress cloud map of the blood vessels with the P-type clip closure. The stress distribution of blood vessels was uniform, with a maximum stress of 0.1857 MPa. The deformation at different positions of the blood vessels clamped by the P-type clamp was relatively uniform. 

**Figure 7:**
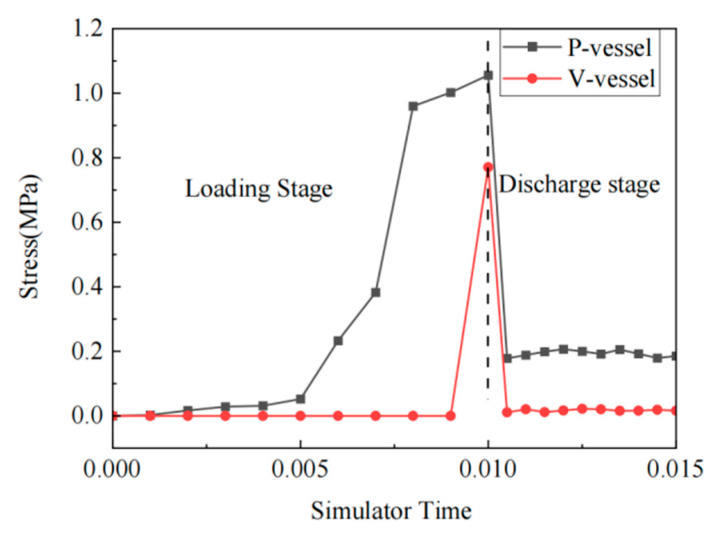
Curve graph of vascular stress changes with simulation time for V-clip and P-clip.

Figure 7 shows the stress curve of the maximum stress point (indicated by the white arrows in Figure 6c,f) of the blood vessels clamped by V-clips and P-clips over time in the simulation program. The vascular clip simulation was mainly divided into two simulation time periods: Loading and Discharge. The stress simulation time of blood vessels clamped by the V-clip and the P-clip was approximately 0.001 and 0.0055, respectively. At 0.015 full release, the stress of V-type vessels was 0.0163 MPa, and the stress of P-type vessels was 0.1594 MPa. The maximum stress unloading pressure of the V-type vascular clip clamping vessel approaches zero. This shows that the V-type clip showed the phenomenon of unloading pressure and rebound. This will cause the vascular clip to be loose, and to easily to fall off. The P-type vascular clip does not have the aforementioned problems and can effectively protect blood vessels. 

**Figure 8:**
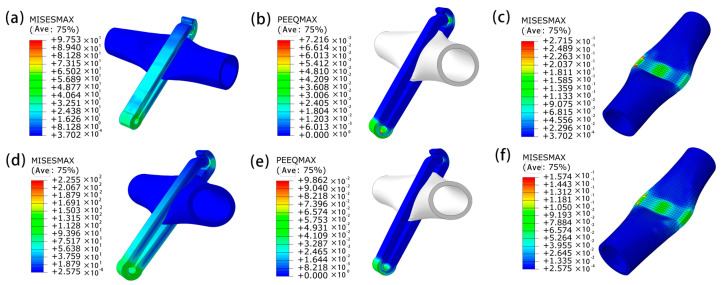
Abaqus simulation analysis of Mg-Zn-Nd (**a**,**b**,**c**), Mg-Zn-Nd-Zr (**e**,**f**) alloy P-clips. Cloud diagram of PEEQMAX (**b**,**e**) and MISESMAX (**c**,**f**) of blood vessels ((**a**): MISESMAX of Mg-Zn-Nd vascular clip; (**d**): MISESMAX of Mg-Zn-Nd-Zr vascular clip).

Figure 8 is a cloud map of the maximum equivalent plastic strain (PEEQMAX) and maximum equivalent stress (MISESMAX) of P-type vascular clips for Mg-Zn-Nd and Mg-Zn-Nd-Zr alloy materials simulated and analyzed by Abaqus. With set simulation parameters, the loading pressure of Mg-Zn-Nd at 200 MPa could ensure the closure of the vascular clip, while the loading pressure of Mg-Zn-Nd-Zr required 280 MPa. The strength and hardness of Mg-Zn-Nd-Zr were better. 

The PEEQMAX of Mg-Zn-Nd-Zr alloy and Mg-Zn-Nd alloy vascular clips in Figure 8 are 0.0928 and 0.0722, respectively. The MISESMAX of Mg-Zn-Nd and Mg-Zn-Nd-Zr alloy vascular clips simulated in Figure 8c,f were 0.2715 MPa and 0.1574 MPa, respectively. The vascular stress of Mg-Zn-Nd-Zr alloy vascular clip was 42.26% lower than that of Mg-Zn-Nd alloy vascular clip. Mg-Zn-Nd-Zr alloy vascular clips had better protection against blood vessels.

**Figure 9:**
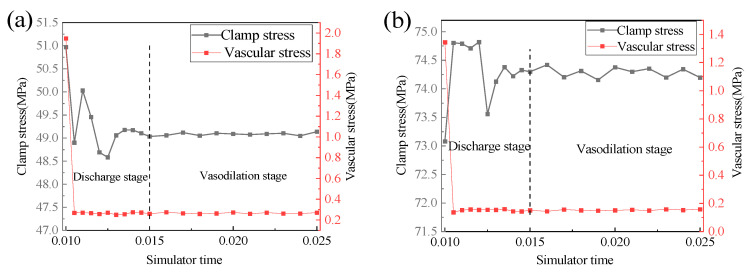
Abaqus simulation analysis of the effect of blood pressure on Mg-Zn-Nd (**a**) and Mg-Zn-Nd-Zr (**b**) alloy vascular clips.

Figure 9 shows the finite element analysis results of using Abaqus to simulate the effect of blood pressure on vascular clip and vascular stress. Simulated blood pressure was 240 mmHg (twice the 120 mmHg blood pressure in the human body). The black curve in the figure shows the stress change of the clamping part of the blood vessels clamp, while the red curve shows the stress change curve at the maximum stress point of the blood vessels. The clamping stress of Mg-Zn-Nd and Mg-Zn-Nd-Zr alloy blood vessels and the clamping stress of blood vessels remained relatively stable under the influence of blood pressure. 

After blood pressure loading, the stress at the fastener position of the Mg-Zn-Nd alloy blood vessel clamp increased by 0.21%, and the blood vessels stress increased by 4.3%. The stress at the fastener position of the Mg-Zn-Nd-Zr alloy vascular clip decreased by 0.09%, and the vascular stress increased by 2.78%. In contrast, the safety of the Mg-Zn-Nd-Zr alloy vascular clip was more stable. 

### 3.4. Corrosion Degradation 

**Figure 10:**
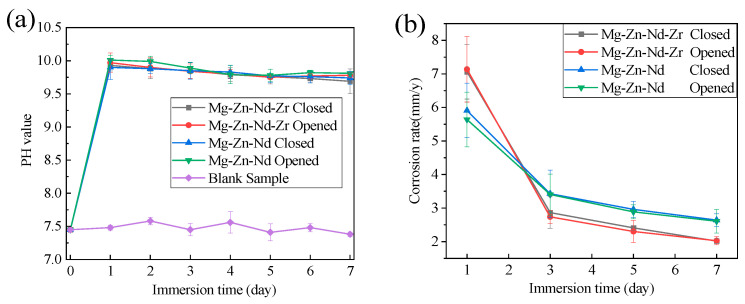
The pH variation (**a**) and weight loss (**b**) of Mg-Zn-Nd-Zr and Mg-Zn-Nd alloy. Vascular clips opening and closing during immersion over 7 days.

Figure 10 shows the pH variation and weight loss rate of Mg-Zn-Nd and Mg-Zn-Nd-Zr alloy vascular clips with open and closed clamps during the 7 days immersion test. Both pH curves were stable between pH 9.5 and 10.1. The pH reached its maximum on the first day and gradually decreased thereafter. The corrosion rates of the Mg-Zn-Nd-Zr and Mg-Zn-Nd alloy vascular clips during the initial immersion period were relatively high (clamp closure rates are 7.06 mm/y and 5.91 mm/y, respectively). As the immersing time prolongs, corrosion products were generated on the surface of Mg alloys. The corrosion products had a certain protective effect on the substrate, leading to a gradual decrease in corrosion rate. Although the trend of corrosion rate change with immersion time was the same, there were still certain differences in corrosion resistance and degradation behavior between the two alloys. 

The corrosion rate of Mg-Zn-Nd-Zr alloy was relatively fast on the first day of immersion (7.14 mm/y), but after the 3rd day the corrosion rate of Mg-Zn-Nd-Zr alloy was significantly lower than that of Mg-Zn-Nd alloy. On the 3rd day, the corrosion rate of Mg-Zn-Nd alloy was 3.43 mm/y, but the corrosion rate of Mg-Zn-Nd-Zr alloy was only 2.74 mm/y (approximately 82.04% of Mg-Zn-Nd alloy). In addition, the corrosion rate of open clips and closed clips of the same alloy composition alloy is similar. Therefore, the stress concentration after the closure of the P-type clip has little effect on the corrosion resistance The design of the arc end structure could reduce the impact of stress on magnesium alloy corrosion.

**Figure 11:**
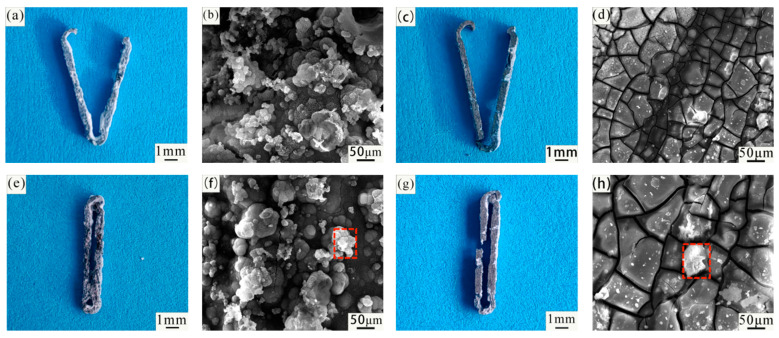
Macroscopic (**a**,**c**,**e**,**g**) and microscopic (SEM) images (**b**,**d**,**f**,**h**) of Mg-Zn-Nd-Zr (**a**,**b**,**e**,**f**) and Mg-Zn-Nd (**c**,**d**,**g**,**h**) vascular clips before and after immersion in SBF for 7 days without acid washing.

Figure 11 shows the macro and micro (SEM) morphology of the Mg-Zn-Nd-Zr and Mg-Zn-Nd alloy vascular clips before and after closure immersed in SBF simulated body fluid for 7 days without pickling. From the macroscopic view, the vascular clip closed by Mg-Zn-Nd alloy had already been disconnected by the 7th day, but the vascular clip closed by Mg-Zn-Nd-Zr alloy remains closed. 

**Figure 12:**
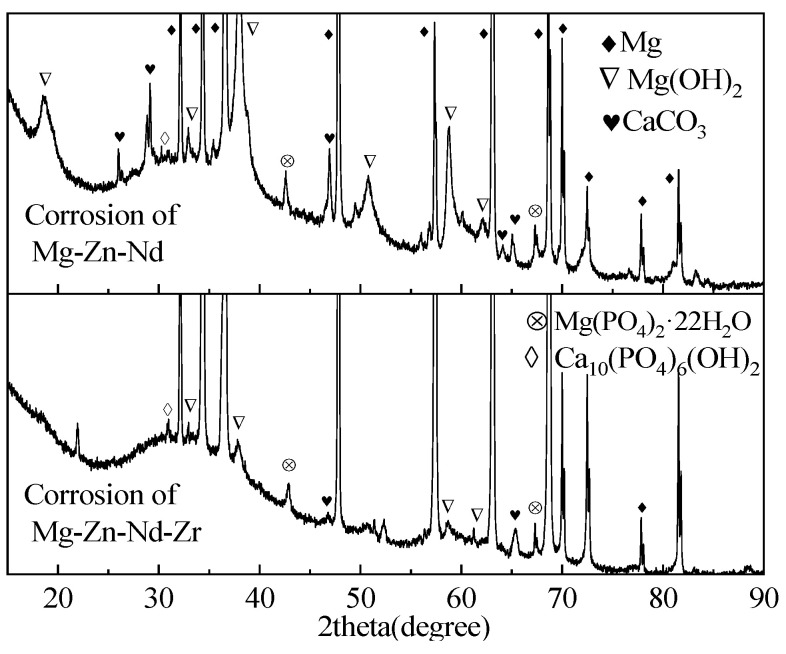
XRD patterns of Mg-Zn-Nd-Zr and Mg-Zn-Nd after 7 days of corrosion.

Figure 11 is corrosion morphology of Mg-Zn-Nd and Mg-Zn-Nd-Zr alloy. The red box in the figure shows the EDS spectrum scanning area. The morphology of the corrosion products of the two alloys is different. The corrosion products of Mg-Zn-Nd-Zr alloy are mostly agglomerated on the surface, while the corrosion products of Mg-Zn-Nd alloy are mostly massive. Table 4 shows the EDS spectrum composition and percentage content of corrosion products in the red boxes in Figure 11f,h. According to the EDS analysis results, the corrosion products of both materials had the same elemental composition, both containing O, Mg, C, P, and Ca. Figure 12 shows the XRD images of two alloy materials after 7 days of corrosion. According to the XRD images, the main components of the corrosion products were Mg(OH)_2_, CaCO_3_, and phosphate. 

**Figure 13:**
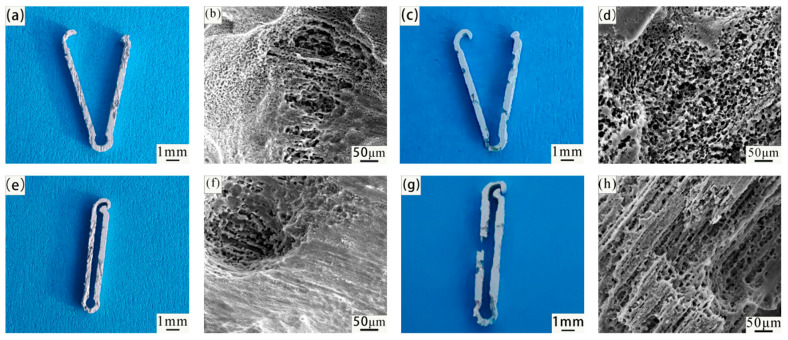
Macroscopic (**a**,**c**,**e**,**g**) and microscopic (SEM) images (**b**,**d**,**f**,**h**) of Mg-Zn-Nd-Zr (**a**,**b**,**e**,**f**) and Mg-Zn-Nd (**c**,**d**,**g**,**h**) vascular clips before and after immersion in SBF for 7 days after acid washing.

Figure 13 is the macro and micro (SEM) morphology of the Mg-Zn-Nd-Zr and Mg-Zn-Nd alloy vascular clips before and after closure soaked in SBF simulated body fluid for 7 days after acid washing. Whether it is closed or not will not affect the degradation behavior, indicating that the improved P-type vascular clip can effectively avoid stress concentration. The composition of the material is the main factor affecting the corrosion resistance. The corrosion degree of Mg-Zn-Nd alloy vascular clips was more severe. Figure 13 is SEM images of the vascular clip after acid washing. The corrosion behaviour of both materials showed pitting.

The corrosion of Mg-Zn-Nd-Zr vascular clips was mainly localized, as shown in the SEM images after pickling with open and closed clamps, and there were obvious pitting pits, but there was no significant corrosion around the pitting pits. The corrosion of the Mg-Zn-Nd vascular clip was different, and the corrosion morphology of Mg-Zn-Nd was relatively uniform, such as in the SEM images after open clamp and closed clamp acid washing, where there was relatively uniform small pitting.

## 4. Discussion

### 4.1. The Influence of Structure on the Application of Vascular Clips 

The stress concentration at the arc end of a general V-type vascular clip was relatively high, resulting in a much faster corrosion rate of the vascular clip compared to the open clamp. Researchers studied the degradation of the general V-type magnesium alloy vascular clip in vitro. On the first day, the degradation rate in vitro was 47.06% faster with closed clips than with open clips. Immersed in SBF solution for the first day, the closed clip corrosion rate was 25 mm/y, and was 5 mm/y on the seventh day, which met the requirements for in vivo experiments [6]. In this experiment, the corrosion rate of the closed clip was only 7.14 mm/y on the first day. In addition, Mao et al. [16] measured, on the 14th day, that the degradation rate in vitro was 33.33% faster with the clamp closed than with the clip open. Multiple experimental results prove that the presence of stress concentration will accelerate the degradation rate of magnesium alloys. Table 5 shows the degradation rate and standard deviation of the modified P-type Mg-Zn-Nd-Zr alloy vascular clip before and after closure. The degradation rate of the closed clip was 1.09% slower than that of the open clip on the first day, and 0.54% faster than that of the open clip on the seventh day. The P-type vascular clip effectively solves the problem that the degradation rate is too fast due to stress concentration.

**Figure 14:**
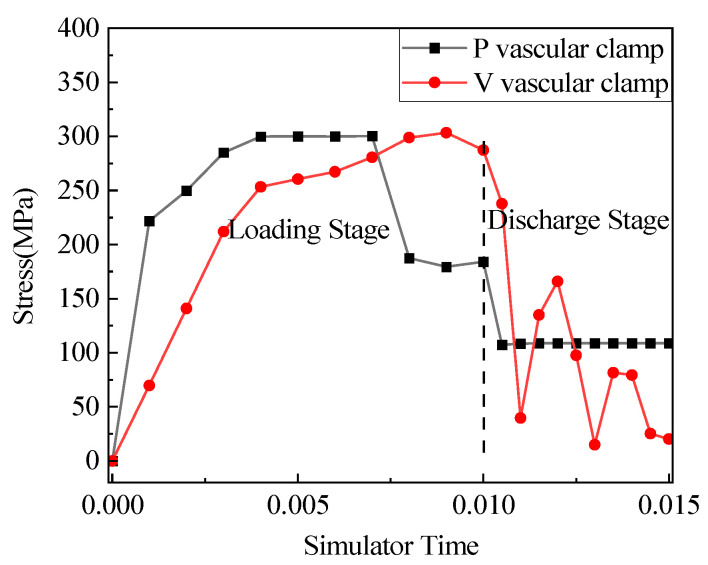
Abaqus simulated curve of stress in V-type and P-type clips. Arc segments with program time.

Figure 14 shows the stress curve of the circular arc segment of Mg-Zn-Nd-Zr alloy closed with general V-type and P-type vascular clips in Abaqus simulation analysis, as a function of simulation program time. During the loading stage, both V-type and P-type structures included the elastic and plastic stages. After the closure of the P-type clip, the stress at the end of the arc would decrease and remain stable. After clamping the blood vessel, the stress at the end of the arc was smaller for the P clip than for the V clip.

The working mode of a regular V-type vascular clip mainly utilizes its own plasticity. This requires high plasticity in the material. If the plasticity of the material itself cannot meet the requirements, it may cause the vascular clip to release force and rebound during the clamping process. After the closure of the V-type vascular clip, the pressure on the blood vessels is uneven, and after the pressure is released, the blood vessels are almost unstressed (as shown in the force curve of the V-type vascular clip in Figure 6g). In the Abaqus simulation process, the distance between the V-type vascular clip and the P-type vascular clip remains consistent. The V-type clip requires a pressure of 480 MPa, while the P-type clip requires 280 MPa. The V-type clip requires greater force to close. Increasing the arc at the bottom of the vascular clip can reduce the stress concentration, but also reduce the plasticity, which will make the vascular clip not tightly closed. Adding a clip part to the top can effectively ensure the closure of the vascular clip. General V-type vascular clips may have gaps after closure due to varying distances between the two arms. The improved P-type vascular clip solves the above problems structurally.

### 4.2. The Effect of Magnesium Alloy Composition on the Performance of Vascular Clips 

The influence of alloying elements on the performance (corrosion resistance and mechanical properties) of vascular clips can be concluded from two aspects, grain refinement and second phase distribution. The different potential of the second phase and matrix leads to galvanic corrosion [23,24,25] The second phase composition of the two alloys is the same. However, the volume fraction of the second phase in Mg-Zn-Nd-Zr and Mg-Zn-Nd alloys is 9.33% and 5.73%, respectively. Therefore, the different performance of Mg-Zn-Nd-Zr and Mg-Zn-Nd alloys is mainly induced by the volume fraction of second phase. The number of the second phase Mg-Zn-Nd-Zr alloys is higher, so the corrosion rate is high due to galvanic corrosion at the initial stage of immersion [7]. The effect of second phase distribution on mechanical properties will not be discussed here, because the precipitation strengthening mechanism is not the main strengthening mechanism in magnesium alloy, and the difference between the second phase volume fraction of two alloys is not obvious enough.

Zirconium (Zr) is an effective grain refiner for magnesium alloys. Zr provides a substrate for heterogeneous nucleation of magnesium grains during solidification, which significantly refines the grains and improves the corrosion resistance of the alloy [26,27]. The addition of Zr in Mg-Zn-Nd alloy can refine the grain size, and the addition of 0.59% Zr in Mg-Zn-Nd alloy can reduce the grain size by 34.09%. The magnesium oxide layer on the alloy is unstable because of the high geometric instability between the magnesium oxide layer and the substrate. After immersion in simulated body fluid (SBF), magnesium oxide will become magnesium hydroxide, resulting in the transformation of crystal type from cubic to hexagonal, and the volume will increase by about two times. It will generate compressive stress in the corrosion product layer, leading to the fracture of the corrosion product. Finer grains will release the compressive stress, making the corrosion product layer more stable and ultimately improving the corrosion resistance [28,29,30,31].

With the addition of 0.59% Zr in Mg-Zn-Nd alloy, the tensile strength of Mg-Zn-Nd alloy increased by 36%. Grain refinement also has a positive effect on the mechanical properties of magnesium alloys. The grain refinement of magnesium alloy will increase the number of grains per unit volume, the number of grains involved in deformation will also increase, and the deformation will become uniform, resulting in large plastic deformation before fracture. At the same time, the total area of grain boundary becomes larger, dislocation barriers become more, and the number of grains with different orientations that need to be coordinated increases, which ultimately makes the strength of Mg-Zn-Nd-Zr alloy stronger [32,33,34].

The magnesium alloy vascular clip needs a certain strength and corrosion resistance. If the strength of magnesium alloy is not enough, it will be easily affected by external forces, such as the deformation of magnesium alloy vascular clip under external forces during clinical use. The corrosion resistance of the vascular clip is the key to ensure the clinical service time. Therefore, the high strength and corrosion resistance of Mg-Zn-Nd-Zr alloy can meet the clinical application requirements of the vascular clip.

## 5. Conclusions

1. The common V-type vascular clip has problems of force release and rebound, high loading pressure, and uneven force distribution during the clamping process. The improved P-type vascular clip solves these problems, and the P-type is more reliable and safer.
2. The tensile strength of Mg-Zn-Nd-Zr alloy is 305.95 MPa, and that of Mg-Zn-Nd alloy is 224.98 MPa. The tensile strength of Mg-Zn-Nd alloy increased by 36% with the addition of 0.59% Zr. The strength of magnesium alloy can improve the stability and safety of vascular clips.
3. The long-term corrosion resistance of Mg-Zn-Nd-Zr alloy is better than that of Mg-Zn-Nd alloy. The Mg-Zn-Nd alloy clip broke and failed on the seventh day, but the Mg-Zn-Nd-Zr alloy remained closed on the seventh day.

## Figures and Tables

**Table 1:** Parameters of Holzapfel arterial wall anisotropic hyper-elastic model [18].

Arterial Layer	C_10_ (kPa)	K_1_ (kPa)	K_2_ (kPa)	φ
Inner layer	55.80	263.66	170.88	60.30
Middle layer	2.54	21.60	8.21	20.61
Outer layer	15.12	38.57	85.03	67.00

**Table 2:** Composition and content of Mg-Zn-Nd and Mg-Zn-Nd-Zr rods (wt.%).

Materials	Si	Fe	Ni	Cu	Zn	Nd	Zr
Mg-Zn-Nd-Zr	0.004	0.004	<0.005	<0.005	1.98	0.54	0.59
Mg-Zn-Nd	0.010	0.003	<0.003	<0.003	1.92	0.53	0.00

**Table 3:** Composition of 1L SBF solution.

NaCl	NaHCO_3_	KCl	K_2_HPO_4_·3H_2_O	MgCl_2_·6H_2_O	HCl	CaCl_2_	Na_2_SO_4_	Tris
8.035 g	0.355 g	0.255 g	0.231 g	0.311 g	39 mL	0.292 g	0.072 g	6.118 g

**Table 4:** EDS spectrum composition and percentage content of Mg-Zn-Nd-Zr and Mg-Zn-Nd vascular clips corrosion after 7 days.

Materials	Percentage	O	Mg	C	P	Ca	Total
Mg-Zn-Nd-Zr	Atomic%	53.75	12.84	12.55	10.42	10.44	100
Mg-Zn-Nd	Atomic%	54.31	8.91	16.85	10.61	9.32	100

**Table 5:** Degradation rate and standard deviation of modified Mg-Zn-Nd-Zr alloy before and after P-type vascular clip (mm/y).

Number of Days	1	3	5	7
Open-clip	7.14 ± 0.98	2.74 ± 0.19	2.30 ± 0.33	2.03 ± 0.13
Closed-clip	7.06 ± 0.81	2.87 ± 0.47	2.41 ± 0.07	2.02 ± 0.07

## Data Availability

The raw and processed data required to reproduce these findings cannot be shared at this time as the data also forms part of an ongoing study.
